# Adaptive evolution of the matrix extracellular phosphoglycoprotein in mammals

**DOI:** 10.1186/1471-2148-11-342

**Published:** 2011-11-21

**Authors:** João Paulo Machado, Warren E Johnson, Stephen J O'Brien, Vítor Vasconcelos, Agostinho Antunes

**Affiliations:** 1 CIMAR/CIIMAR, Centro Interdisciplinar de Investigação Marinha e Ambiental, Universidade do Porto, Rua dos Bragas, 177, 4050-123 Porto, Portugal; 2 Instituto de Ciências Biomédicas Abel Salazar (ICBAS), Universidade do Porto, Portugal; 3 Laboratory of Genomic Diversity, National Cancer Institute, Frederick, MD 21702-1201, USA; 4 Departamento de Biologia, Faculdade de Ciências, Universidade do Porto, Rua do Campo Alegre, 4169-007 Porto, Portugal

## Abstract

**Background:**

Matrix extracellular phosphoglycoprotein (MEPE) belongs to a family of small integrin-binding ligand N-linked glycoproteins (SIBLINGs) that play a key role in skeleton development, particularly in mineralization, phosphate regulation and osteogenesis. MEPE associated disorders cause various physiological effects, such as loss of bone mass, tumors and disruption of renal function (hypophosphatemia). The study of this developmental gene from an evolutionary perspective could provide valuable insights on the adaptive diversification of morphological phenotypes in vertebrates.

**Results:**

Here we studied the adaptive evolution of the MEPE gene in 26 Eutherian mammals and three birds. The comparative genomic analyses revealed a high degree of evolutionary conservation of some coding and non-coding regions of the MEPE gene across mammals indicating a possible regulatory or functional role likely related with mineralization and/or phosphate regulation. However, the majority of the coding region had a fast evolutionary rate, particularly within the largest exon (1467 bp). Rodentia and Scandentia had distinct substitution rates with an increased accumulation of both synonymous and non-synonymous mutations compared with other mammalian lineages. Characteristics of the gene (e.g. biochemical, evolutionary rate, and intronic conservation) differed greatly among lineages of the eight mammalian orders. We identified 20 sites with significant positive selection signatures (codon and protein level) outside the main regulatory motifs (dentonin and ASARM) suggestive of an adaptive role. Conversely, we find three sites under selection in the signal peptide and one in the ASARM motif that were supported by at least one selection model. The MEPE protein tends to accumulate amino acids promoting disorder and potential phosphorylation targets.

**Conclusion:**

MEPE shows a high number of selection signatures, revealing the crucial role of positive selection in the evolution of this SIBLING member. The selection signatures were found mainly outside the functional motifs, reinforcing the idea that other regions outside the dentonin and the ASARM might be crucial for the function of the protein and future studies should be undertaken to understand its importance.

## Background

Dentin, one of the major mineralized tissues of teeth, is deposited by odontoblasts, which synthesize collagenous and non-collagenous proteins (NCPs) [1,2]. Among the NCPs, there is a family of small integrin-binding ligand N-linked glycoproteins (SIBLINGs) consisting of dentin matrix protein 1 (DMP1), dentin sialophosphoprotein (DSPP), integrin-binding sialoprotein (IBSP), matrix extracellular phosphoglycoprotein (MEPE, also known as OF45) and osteopontin (SPP1) [3]. These genes share common genetic and structural features, including a small non-translational first exon, a start codon in the second exon and a large coding segment in the last exon (although exon number varies among the different genes) [4]. The entire SIBLING protein family likely arose from the secretory calcium-binding phosphoprotein (SCPP) family by gene duplication, since this cluster of genes encodes proteins with similar molecular-structural features and functions [5].

Members of this gene family are encoded by a compact tandem gene cluster (located on chromosome 4 q in Human and 5 q in mouse) characterized by: (*i) *common exon-intron features, (*ii*) the presence of the integrin-binding tripeptide Arg-Gly-Asp (RGD) motif that mediates cell attachment/signaling via interaction with cell surface integrins [4], and (*iii*) post-translational modifications of conserved phosphorylation and N-glycosylation sites [4]. In humans, the MEPE protein (525 amino acids) is encoded by four exons with a 1960 bp transcript with two N-glycosylation motifs (at residues 477-481), a glycosaminoglycan (SGDG) attachment site at residues 256-259, and the RGD cell attachment motif at residues 247-249 [6]. The RGD motif has a similar function in other members of the SIBLING's (DSPP, DMP1, IBSP, and SPP1) [7]. The protein MEPE has several predicted phosphorylation sites/motifs for protein kinase C, casein kinase II, tyrosine kinase, and cAMP-cGMP-dependent protein kinase and a large number of N-myristoylation sites that appear to be also a feature of the RGD-containing proteins [7]. The acidic serine-aspartate-rich MEPE-associated motif (ASARM motif) occurs at the C-terminus in MEPE (residues 509 to 522) [7] and when phosphorylated this small peptide can bind to hydroxyapatite and inhibit mineralization [8].

The basic MEPE protein was first cloned from a tumor resected from a patient with tumor-induced osteomalacia (OHO) [7,9], which is associated with hypophosphatemia and is caused by a renal phosphate wasting. The MEPE gene is also up-regulated in X-linked hypophosphatemic rickets (XLH or HYP-osteoblasts) and OHO-tumors [7,10-14]. Under normal conditions it is expressed primarily in osteoblasts, osteocytes, and odontoblasts [13].

Targeted disruption of the MEPE gene in mouse causes increased bone formation and bone mass, suggesting that MEPE plays an inhibitory role in bone formation and mineralization [15]. In humans, MEPE inhibits mineralization and is also involved in renal phosphate regulation [16,17]. The inhibition of mineralization and phosphate uptake are related with the protease resistant small peptide ASARM motif located near the end of the protein [7,17]. However, the MEPE protein has dual functions depending on the proteolytic processing. When the protein is cleaved by cathepsin B or D into several fragments, the small peptide ASARM is released [18] and when phosphorylated, this small peptide can bind the hydroxyapatite crystal and inhibit mineralization [8]. By contrast, when fragments containing the RGD motif are released and the ASARM is not degraded by proteases, mineralization is accelerated [19]. The influence of MEPE-ASARM peptides in the modulation of mineralization is due to a protein-protein interaction with PHEX, an X-linked phosphate-regulating endopeptidase homolog (also called the minhibin model) [17]. PHEX is also expressed in osteoblasts, osteocytes and odontoblasts and the protein interacts with MEPE, protecting it from the proteolytic process (from cathepsin-B) and preventing ASARM from being released into blood circulation [8]. Most of the disorders associated with MEPE result from a malfunction of this PHEX-MEPE interaction, which in turn leads to an increase of ASARM blood levels.

The majority of mammalian genes are strongly conserved in the coding sequence [20,21]. Genes carrying signatures of selection may be involved in adaptation and functional innovation, and often have elevated ratios of nonsynonymous/synonymous nucleotide substitutions (dN/dS) in their coding regions [22]. However, evolutionary rates of nuclear and mitochondrial genes are not equal in all the mammalian lineages [23]. For example, while rodents tend to accumulate more mutations in nuclear genes than humans [24], the differences between the rates in the two lineages seems to be smaller than the generation time difference [23].

Since MEPE protein has an important role in the regulation of the skeleton mineralization process and since the mineralized tissue is a critical innovation in vertebrate evolution, the evolutionary study of this developmental gene could provide valuable insights on the adaptive diversification of morphological phenotypes in mammals. As the MEPE gene has been suggested to be under selection [25], our objective was to undergo a thorough analysis to evaluate signatures of positive selection using both a gene-level and protein-level approaches. We assessed the evolution of the MEPE protein-coding gene in 26 mammalian species, from Hyracoidea to Primates, showing that while four regions/motifs in the MEPE gene have a high degree of conservation, the majority of the coding region has a fast evolutionary rate, especially in rodents and tree shrews. Indeed, evidence of strong positive selection (gene and protein-level) was found in 20 amino acids that encompass MEPE protein, highlighting the role of molecular adaptation in the functionality of this gene.

## Results

### Presence of the MEPE in vertebrates

Twenty-six mammalian MEPE sequences were retrieved from the GenBank and Ensembl databases, comprising eight different mammalian Orders (Additional file 1: Table S1). In addition, sequences of the putative MEPE orthologue, Ovocleidin-116, were obtained from the available bird genome projects (*Gallus gallus, Taeniopygia guttata, Meleagris gallopavo*) for comparative purposes. For the majority of the mammals considered in this work, the MEPE gene encompasses four exons that encode a transcript that varied from 1272 bp in *Ochotona princeps *to 2030 bp in *Pan troglodytes*. Some of the smallest reported transcripts may be incomplete, as in the case of *O. princeps*, which is missing a stop codon. The absence of the ASARM motif in the MEPE's C-terminal in some species (*Equus caballus*, *Ochotona. princeps, Otolemur garnetti and Pteropus. vampyrus*) also suggests that those genes were not fully annotated. Thus, we performed a detailed search in databases for those species using TBLASTN [26], which led to the identification of the ASARM in *E. caballus*, but not in *O. princeps*, *O. garnetti *and *P. vampyrus *(in these cases, the missing end portion of the protein corresponds to the end of the contig available in the database). However, several stop codons are present between the end of the present sequence and the putative ASARM motif in the *E. caballus *sequence and therefore it was not included in subsequent analyses.

**Figure 1:**
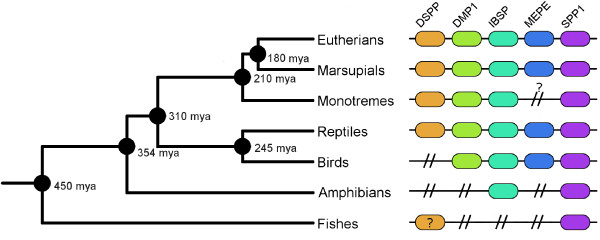
**SIBLING (DSPP, DMP1, IBSP MEPE and SPP1) presence in vertebrates**. Illustrative representation of the SIBLING (DSPP, DMP1, IBSP MEPE and SPP1) genes presence/absence in vertebrates. The estimated divergence time of the different groups are placed near the nodes.

We performed blast searches (TBLASTN and TBLAST) to determine if MEPE is present in non-mammalian or non-avian vertebrates (such as fish and amphibians), but we were not able to detect an orthologue in those lineages, suggesting that this gene may be considerably differentiated or even absent. In chicken (*G. gallus)*, a similar protein has been already described, MEPE/OC116 [27] (i.e. Ovocleidin 116), and it is likely a homologue of MEPE. This orthologue is also present in two other birds (*T. guttata, M. gallopavo*). Although our initial BLAST searches did not return a significant hit in reptiles, a recent study suggests the presence of MEPE in *Anolis carolinensis *[28]. Blast searches for the MEPE gene in teleost fishes (e.g. *Takifugu rubripes*, *Oryzias latipes *and *Danio rerio*) did not retrieve a significant hit. Even searching synteny blocks between Human and Zebrafish (results not shown), did not provide evidence of MEPE. This result is concordant with previous studies [5,29-32] that show the likely presence of two genes belonging to the SIBLING family in teleost fishes but not a MEPE orthologue. Mammals and reptiles are the only tetrapod lineages with all five SIBLING family genes (Figure 1), as previously suggested [28,29].

### Sequence analyses

**Figure 2:**
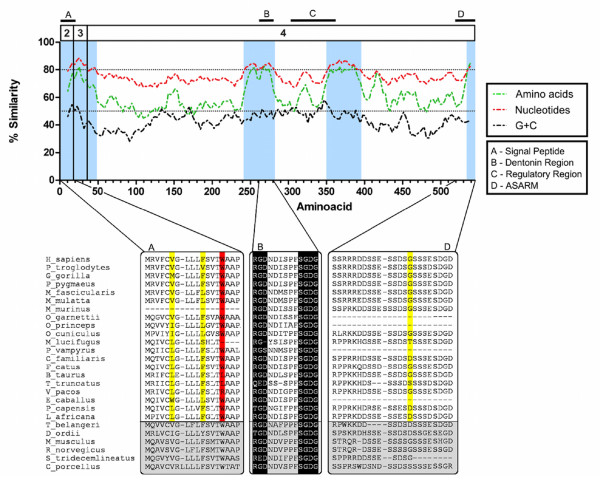
**Sliding window plot and motifs comparison of MEPE across the 26 mammalian species**. Sliding window plot of GC-content and nucleotide and amino acid conservation among the 26 mammalian MEPE coding sequences (exons 2, 3 and 4) that were used in this evolutionary study. The plot was calculated after pairwise deletion of ambiguous sites and the windows were adjusted to correspond to the same scale. The blue shading identifies conserved regions (> 80% nucleotide or amino acid similarity), the red line tracks nucleotide similarity, the green line amino acid similarity and the black line % GC content. The three motifs/regions are represented within the boxes A-Signal Peptide-, B-Dentonin (SDGG, RGD), C- Putative regulatory regions and D-ASARM. The yellow and red shadow represents selection at codon level and amino acid level, respectively, while the grey shadow correspond to the species excluded from the positive selection analyses at site level.

At the protein level MEPE is highly variable, especially in the region encoding the last exon, with pairwise amino acid similarity among mammals varying from 99% to 28%. Nevertheless, four important regions within MEPE had high amino acid conservation (> 80%): the signal peptide, the RGD and SGDG regions (the glycosaminoglycan attachment site), and the ASARM motif (Figure 2). Moreover, the protein is also highly conserved from positions 887 to 1091 bp of the human sequence, a region associated with a putative regulatory region (Ensembl annotation). Exon 2, only 54 bp long, encodes mainly the signal peptide and is highly conserved. Remarkably, two alanines (hydrophobic residues) are conserved in 25 of the 26 mammalian species studied (Figure 2). The fourth exon (that encodes most of the protein) comprehends the RGD, SGDG, and ASARM motifs and the putative regulatory region. GC content was similar along most of the coding sequences, with a few segments above 50% (Figure 2).

**Figure 3:**
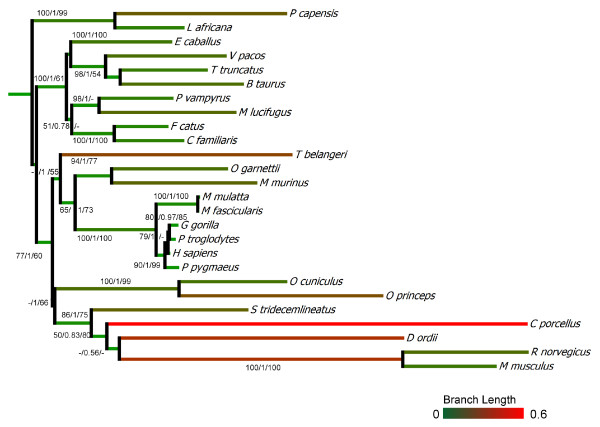
**Phylogenetic tree of MEPE**. Depiction of MEPE protein phylogeny constructed using Bayesian inference (Bayes), Maximum Likelihood (ML) and Neighbor-Joining (NJ) algorithms. Support for each node is summarized on the branch prior to the node (ML/Bayes/NJ). For the NJ and ML analysis the bootstrap values < 50 are represented with the symbol (-). Branches are shaded with a gradient based on the branch length, from green (short) to red (longer).

Phylogenetic analyses of the mammalian MEPE protein sequences showed similar overall topologies with the three reconstruction methods used: Neighbor-Joining (NJ), Bayesian (BY), and Maximum Likelihood (ML) (Figure 3). The topologies were also consistent with those retrieved when using the MEPE nucleotide sequences (results not shown), and all were mostly compatible with the accepted phylogeny of mammals [33-36]. However, Rodentia and Scandentia had long branches, suggesting higher mutation rates (increased number of synonymous and non-synonymous substitutions). We performed the two-sided Kishino-Hasegawa test (KH), the Shimodaira-Hasegawa test (SH), and the Expected Likelihood Weights (ELW) in TREE-PUZZLE to determine the best-fitting tree. The test of the three resulting phylogenetic trees suggests that the ML tree best fit the multiple sequence alignment (values of KH and SH were 1, and therefore were highly significant and ELW = 0.7771), although the Bayesian tree was not significantly worse than the ML tree (Additional file 2: Table S2). Conversely, after removing the rodents and tree shrew the three methods produced similar topologies and therefore no significant differences were obtained in the tests implemented in TREE-PUZZLE. The best-fitting trees for the two alignments were then used in subsequent analyses. Likelihood mapping, implemented in TREE-PUZZLE to inspect the phylogenetic signal of the alignment (Additional file 2: Table S2), showed a relevant value for both alignments that was slightly reduced when rodents and tree shrew were included. Phylogenies based only on transversions or only on the first and the second coding positions showed the same patterns (data not shown).

**Figure 4:**
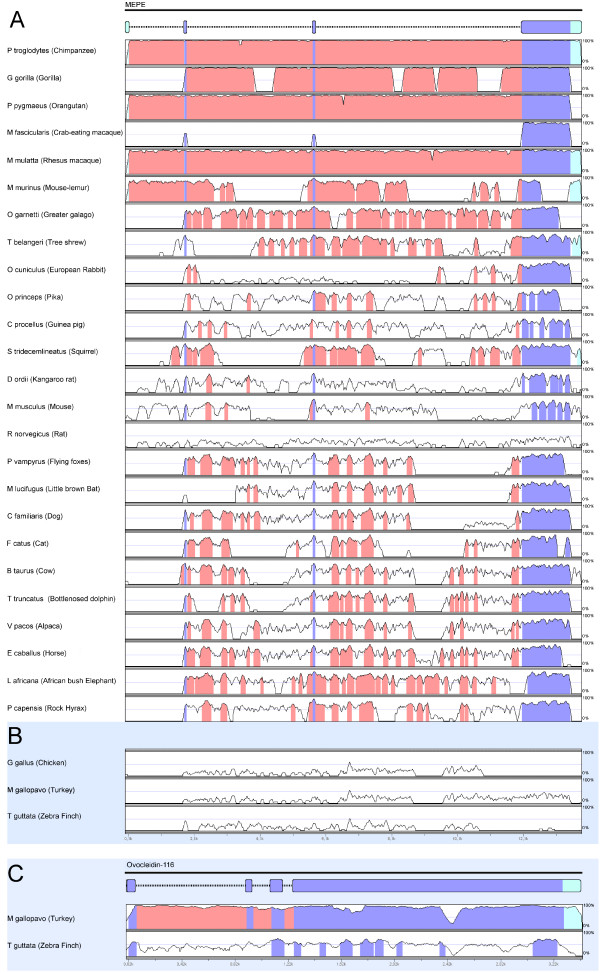
**Nucleotide conservation of MEPE in mVISTA**. (A) MEPE gene conservation between 25 mammalian species orthologues compared with the human MEPE sequenced portrayed in an mVISTA plot with the 100 bp window with a cut-off of 70% similarity. The Y-scale represents the percent identity ranging from 0 to 100%. (B) Human MEPE compared with the three bird orthologues. (C) Pairwise comparison of the two birds ovocleidin-116 with the *G. gallus *orthologue. Exons are highlighted in blue, nontranslated regions in green-blue, and conserved non-coding sequences (CNS) in pink.

In the non-coding gene regions, the nucleotide similarity plots illustrate that the human sequence is highly conserved relative to the other primates, *Pan troglodytes*, *Gorilla gorilla *and *Macaca mulatta *(Figure 4A). At a lower level the comparison of the MEPE non-coding regions across all species showed several Conserved Non Coding Sequences (CNS) after pairwise comparisons with the human sequence across all species. This intronic conservation is particularly important since CNS have been associated with transcriptional regulation [37]. The length of CNS decreases when the Human MEPE is compared with homologues from more distantly related species, but not necessarily in a direct association with phylogenetic distance (Figure 4A). For instance, the dog (*Canis lupus familiaris*) and cattle (*Bos taurus*) are phylogenetically more distant from human than the mouse (*Mus musculus*) and rat (*Rattus norvegicus*), but showed a higher conservation both in coding and non-coding regions of the gene (Figure 4A). By contrast, in the Order Lagomorpha there is less conservation in the intronic regions but high conservation in the coding regions, and in rodents, there are high numbers of differences both in coding and non-coding regions (Figure 4A). As expected, birds showed low similarity in both coding and non-coding region with mammals (Figure 4B), although they exhibited high similarity in the coding regions in pairwise comparisons with *G. gallus *(Figure 4C). Furthermore, the two Galliform species also were similar in the non-coding regions while the *G. gallus *and the *T. guttata *did not present high intronic conservation.

**Figure 5:**
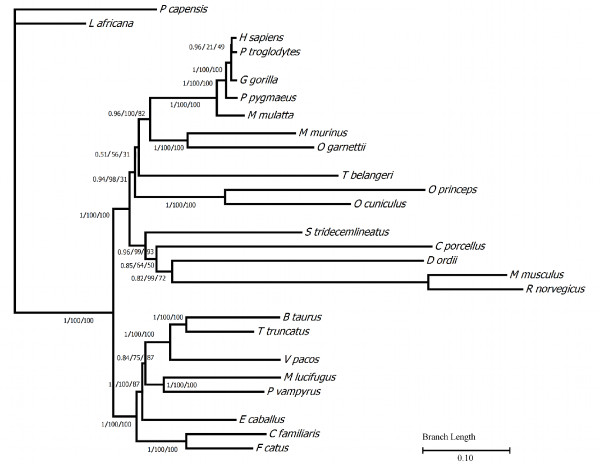
**Phylogenetic tree of MEPE intronic regions**. Phylogenetic depiction of the MEPE intronic region tree reconstructed using Bayesian inference (Bayes), Maximum Likelihood (ML) and Neighbor-Joining (NJ) algorithms. The labels are positioned near the branches supporting the tree and inside the brackets (Bayes/ML/NJ). The methodology was similar to the implemented in the coding regions. The alignment of the intronic regions comprehends 856 out of 21120 sites completely clean of gaps in all the species (except for the *Macaca mullata *since the intronic regions was not available).

Given the large difference in average length of CNS (from 1.8 kb in Lagomorpha to 8.4 kb in Primates) and their high similarity (from 71.4% in Lagomorha to 89.5% in Primates) (Additional file 3: Figure S1), it is not surprising that introns have ample phylogenetic signal for gene-tree reconstruction. The alignment of the intronic regions comprehends 21120 bp and 856 of those sites were clean of ambiguity data in all the species (*Macaca mullata *was excluded since the intronic regions were not available). MEPE intronic sequences provided a significant phylogenetic signal across all the studied mammals, resulting in similar topologies as those trees reconstructed from coding regions and protein suggesting an appreciable level of evolutionary constraints in MEPE introns (Figure 5).

**Figure 6:**
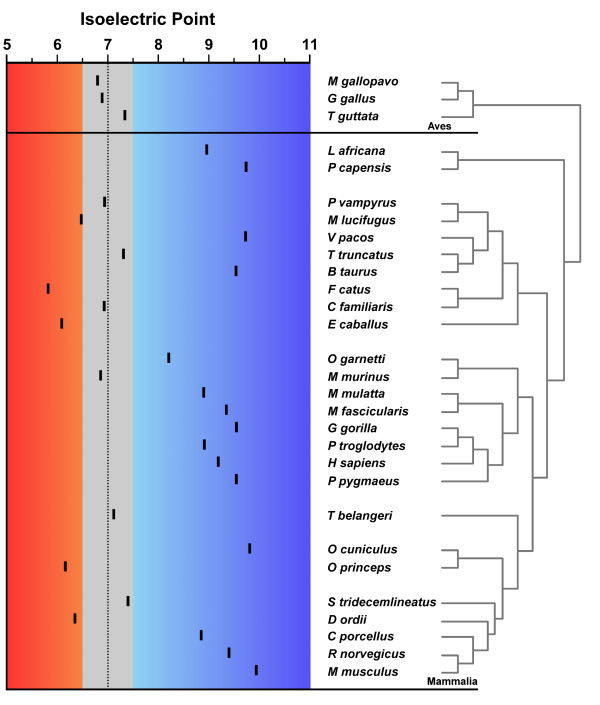
**MEPE isoelectric points (pI) calculated for the 26 mammalian and 3 avian species**. The red shadow represents the acid pI while the blue the basic pI, the grey shadow shows the nearly neutral proteins, from 6.5 to 7.5.

The MEPE protein is generally basic, with an average Isoelectric Point (pI) of 8.20 in the mammal species studied. Generally the pI was lower in Laurasiatheria, reaching 5.82 in *Felis catus *(Figure 6). In the three available avian sequences pI was less than 7 in the two Galliformes and slightly above 7 in Passeriformes. These differences in pI may have dramatic effects on the protein folding, as those changes are caused by significant differences in the polarity of the amino acids that compose the protein.

### Functional motifs

The cell attachment region, RGD, situated near the center of the MEPE protein, is fully conserved in 20 of the 26 mammalian species (Figure 2). However, some changes are observed in *Tursiops truncatus*, *Procavia capensis*, the bats *Pteropus vampyrus *and *Myotis lucifugus*, and in the rodents *Dipodomys ordii *and *Spermophilus tridecemlineatus *(Figure 2) and it is likely that such amino acids changes in the RGD motif may have functional relevance. Moreover, the RGD motif is also present in other genes of this gene-cluster family.

The SDGD is completely conserved among all the mammals, reinforcing the premise that this peptide region is, along with RGD, important to the MEPE function. These two motifs constitute the dentonin region, which was not detected in any of the others members of the SIBLING protein family. The chicken and the turkey MEPE orthologues appear to be exceptions, since they do not have the cell-adhesion motif, RGD, but contain the glycosaminoglycan-binding motif, SGDG. In these species we found a HGD near the SGDG motif, suggesting that RGD is replaced by HGD (Additional file 4: Figure S2). A similar change from RGD has been described in other members of the DSPP orthologues (e.g. in rat, *Rattus norvegicus*, the RGD replaces the HGD) [38]. Nevertheless, in zebra finch (*T. guttata*) we found the RGD motif but not the SGDG region (Additional file 4: Figure S2). The ASARM motif is highly conserved within the 21 mammals for which ASARM is annotated (average above 85%), although the Bottlenose dolphin (*Tursiops truncatus*) has a similarity of only 59.1%. Pairwise similarity among birds was 79.9% (among the three avian species), but on average only 27.3% similarity was observed between birds and the mammalian ASARM. Moreover, in birds this motif is capped at the C-terminal by 21 (*G. gallus, M. gallopavo*) to 24 (*T. guttata*) amino acids, and this region shows 77.2% similarity between *G. gallus *and *M. gallopavo *but less than 40% between these two species and *T. guttata*, showing that this region in birds is probably less constrained than the ASARM.

### Rodentia and Scandentia selection signatures

**Figure 7:**
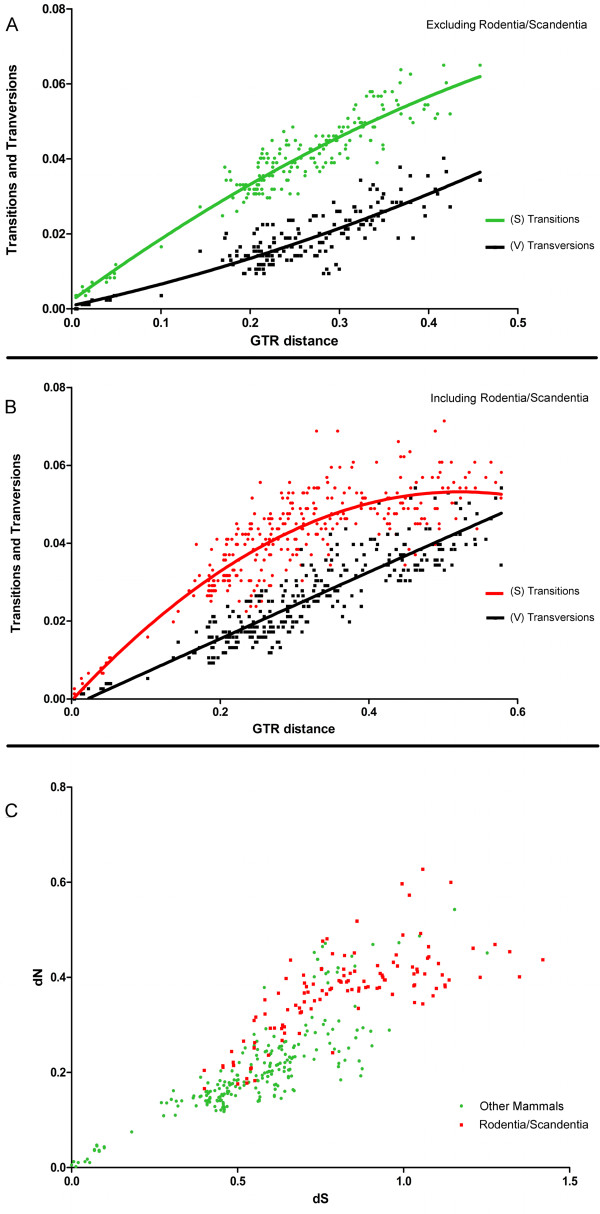
**Accumulation of saturation and altered evolutionary rate in Rodentia and Scandentia compared with other mammals**. (A) Nucleotide saturation plots excluding rodents and the tree shrew, showing transitions (S) and transversions (V) accumulated in the third position; and the same analysis (B) including the rodents and the tree shrew (C) Pairwise dN/dS comparison of rodents and the other mammals.

The saturation plots (Figure 7A and 7B) showed that the rodents and the tree shrew have accumulated a very high number of transitions and transversions relative to other mammalian species (also apparent in the long branches of those species in the phylogenetic tree; Figure 3). Saturation of synonymous mutations can bias the analysis of positive selection due to an underestimation of dS that will increase ω [39]. Therefore, these species have been excluded from the codon and amino acid properties selection analyses (site models). When we grouped rodents and the tree shrew, and compared them with the other mammals, the Relative Ratio Test (RRT) [40] showed that MEPE accumulated more mutations in the orders Rodentia and Scandentia (Table 1), with an average number of synonymous substitutions of 0.635 and non-synonymous substitutions of 0.304, in contrast with the other mammalian species with 0.527 and 0.235, respectively (both analyses being highly significant; p < 0.025). The tree shrew and the rodents compared with the others mammals, had a higher GC percentage (49.9% versus 44.9%, respectively). This shows that Rodentia and Scandentia have accumulated more synonymous and non-synonymous substitutions (Figure 7C), which is consistent with the phylogenetic analyses that suggest that the rodents and the tree shrew have an accelerated rate of evolution.

**Table 1:** Results from the RRTree test comparing substitution rates in Rodentia, Scandentia and the other mammals.

Group	%GC	Ka	Ks
Rodentia and Scandentia (n = 6)	49.9	0.304	0.635
Other Mammals (n = 20)	44.9	0.235	0.527
p-value		< 0.01	0.025

**Figure 8:**
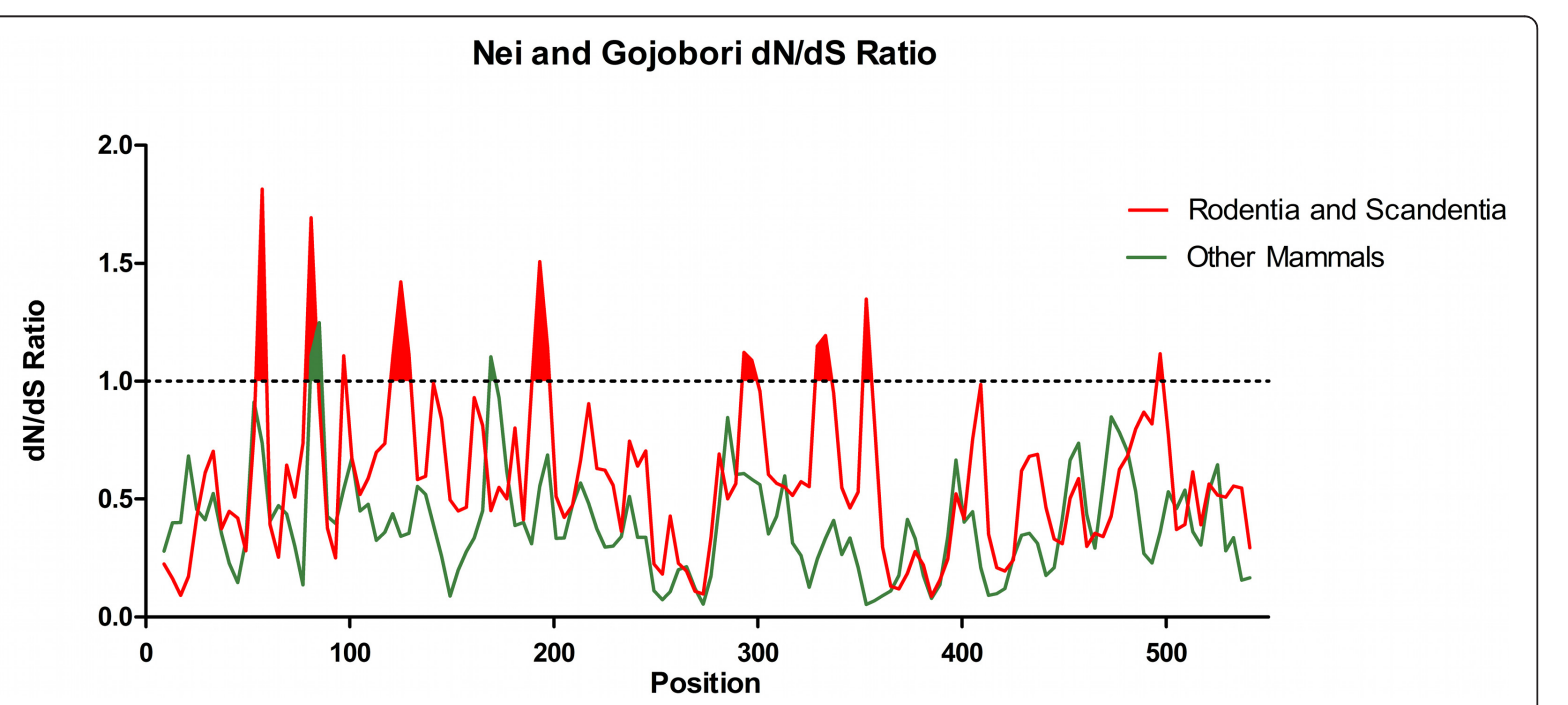
**Differences in the selection pattern in Rodentia and Scandentia compared with other mammals**. Sliding window analysis of the Ka/Ks ratio applying the Nei and Gojobori method for the rodents and tree shrew MEPE compared with the other mammalian species.

To evaluate if orders Rodentia and Scandentia have different sites under positive selection we compared the branch-site model A using the rodents (5 sequences) and tree shrew (1 sequence) as the foreground branch versus the other mammals as background branch (Additional file 5: Table S3). The rodents had 12 sites under positive selection, with four of these being highly significant (PP > 0.95) after the Bayes Empirical Bayes (BEB) analysis; 42-Tyr, 158-Lys, 239-Gly, 247-Asp (using the *Mus musculus *protein as reference). The likelihood ratio test (LRT) demonstrated that the branch-site analysis was statistically significant (p < 0.04). Sliding window analysis using the Nei-Gojobori method also presented significant differences in the sites/regions under positive selection between the rodents/tree shrew and the other mammals (Figure 8). When we applied a window = 15 and step = 9, the rodents and the tree shrew showed eight regions with a dN/dS > 1, while the others species had only two regions > 1, suggesting that the rodents not only present an accelerated rate of evolution but also exhibited a different selection pattern in the protein (Figure 8).

### Selection signatures at the codon level

The codeml test implemented in PAML was used to compare five different nested models in two situations, i.e. including or excluding the ambiguity data in the alignment. The MEPE protein had a global dN/dS ratio of 0.462, with 75 sites under negative selection and 17 sites under positive selection (Model 8 not removing the ambiguity data). When ambiguous data was removed the LRT's for the nested models, M1-M2, was rejected (Table 2), so the results of positive selection for M2 were not taken into account. In the LRT comparison between the more parameter-rich nested pairs of models (M8-M7), twice the log-likelihood difference was 7.1717(Table 2), rejecting M7 and favoring M8 (Chi-square df = 2; p < 0.05). Under M8, 87% of the sites fit the β distribution (1.584- 2.275), but 13% of the sites had a ω_1 _= 1.30. For posterior probabilities of ω > 1 using BEB with M8 vs. M7, nine sites were under positive selection (Table 2). However, none of these sites passed the stringent criterion of statistical significance PP > 0.95 (using the method BEB as the statistical post-analysis). Additionally, the LRT between the M8 and the alternative null model M8a was 1.95, below the critical value (2.71 at p < 0.05), and therefore not favoring the evolutionary model. However, it has been shown that in some cases this alternative LRT test has less power when the category of positively selected sites has a ω value that is only slightly larger than one [41].

**Table 2:** PAML results of MEPE for the 20 mammalian species (excluding ambiguity data).

Model	Parameters	LnL	Test	LRT
Model 0	ω = 0.46086	-7362.999747		

Model 1	p_0 _= 0.63812 p_1 _= 0.36188	-7308.340814		

Model 2	ω_0 _= 0.27631 ω_1 _= 1.00000 ω_2 _= 1.00000			
	p0 = 0.63812 p_1 _= 0.27244 p_2 _= 0.08944	-7308.340814	M2 vs M1	0

Model 7	p = 1.06299 q = 1.09727			
		-7306.257659		

Model 8	p_0 _= 0.86974 p = 1.58369 q = 2.27462			
	(p_1 _= 0.13026) ω_1 _= 1.29913	-7302.671784	M8 vs M7	7.1717

The evaluation of positive selection using the model implemented in Single Likelihood Ancestor Counting (SLAC) showed three sites under selection, one of those sites being similar to that retrieved with model M8 in PAML. Since SLAC tends to be quite conservative, we also estimated the selection signatures using the Fixed Effects Likelihood (FEL) model, which is assumed to be more powerful than SLAC [42,43]. The FEL model revealed a total of 23 sites under selection using this model, including a mutation in the highly conserved small peptide ASARM (from aspartate to glicine) (Figure 2). Such a radical change in ASARM was only observed in a few species and further studies are needed to better document its frequency across mammals. All the sites presented in the model implemented in Datamonkey have a significance value (p < 0.10) in FEL and SLAC, which is an accepted level of significance for the test of those models [42]. When we use a significance threshold of 0.05, the number of positive selected amino acids decreased to 14 in FEL and zero in SLAC, meaning that 9 sites (out of the 23 detected with a significance level of 0.10) had less evidence of being under strong positive selection. However, these positions may still be indicative of selection signatures. Recombination can affect several analyses, including phylogenetic reconstruction and analysis of positive selection [44]. Therefore, we assessed gene recombination using GARD implemented in the Datamonkey web-based server [43] and repeated the selection analysis including and excluding recombination in the dataset. Partitioning the data did not change the conclusions of the positive selection analyses (data not show), suggesting that recombination is not significantly affecting the MEPE gene evolution.

No additional sites were found using the SLR [45], but six sites under selection in the previous analysis were also statistically supported. Overall, across MEPE, 32 of the 525 sites (referenced to the length of the human MEPE) were under positive selection; additionally six sites were supported by more than one codon analysis (9 in PAML, 23 in FEL, 3 in SLAC, and 6 in SLR) (Additional file 6: Table S4).

### Selection at the amino acid level

Selection models that use dN/dS ratios to detect selection are generally not sensitive enough to detect subtle molecular adaptations [46]. It is therefore necessary to employ alternative criteria within generally conserved protein-coding genes or within proteins with strict motifs intermixed with regions under fast directional evolution. Therefore, we used TreeSAAP [47], which evaluates destabilizing radical changes at each site, and an empirical threshold of change in three properties was applied as evidence that a site is under positive (or negative) selection.

At the global protein level, eight of 31 amino acids properties were under strong positive selection in MEPE (p < 0.001 for five and p < 0.05 for the remaining three properties) (Table 3). Remarkably, pI is one of these eight properties under positive selection in MEPE which may also explain the high variability in pI observed across taxa (Figure 6).

**Table 3:** MEPE properties under positive selection determined in TreeSAAP.

Property	Category	Z-Score
Compressibility	7	3.783***
Equilibrium constant (ionization of COOH)	8	3.236***
Isoelectric point	8	3.418***
Power to be at the C-terminal	7	1.926*
Power to be at the middle of alpha-helix	7	3.757***
Power to be at the N-terminal	8	2.373**
Solvent accessible reduction ratio	7	3.953***
Turn tendencies	7	2.307*

List of properties under selection, the impact category and the level of significance (*** p < 0.001; ** p < 0.01; * p < 0.05).

At the amino acid site level, MEPE has 181 sites (33.8%) under positive selection in at least one property. Although applying the empirical threshold of at least three properties showing signatures of positive selection the number of sites is reduced to 41 (7.6%) (Additional file 7: Table S5). The majority of these 41 sites are located in the N-terminal region of the protein and the dentonin region (68% of the positive selected sites). The alternative calculation method was performed using CONTEST and estimates of variation in amino acid charge and volume revealed 79 sites with signatures of positive selection for at least one of the amino acid properties (Additional file 8: Table S6). However, after the Bonferroni and False Discovery Rate (FDR) correction, only one site showed positive selection. This site, located at position 354 in the alignment (position 349 in the human sequence), corresponds either to lysine or glutamate and was not detected by TreeSAAP. The ancestral protein reconstruction in TreeSAAP, based on the baseml implemented in PAML, shows that glutamate is present in the common ancestor of non-Afrotheria mammals, suggesting that the radical change to lysine occurred in Cetartiodactyla, Perissodactyla and in at least one representative of the Lagomorpha.

Based on selection analyses at the protein level across MEPE, 42 of the 525 sites (human MEPE as reference) were under selection at the amino acid level (41 detected with TreeSAAP and 1 with CONTEST).

### Selection at codon and amino acid level

**Figure 9:**
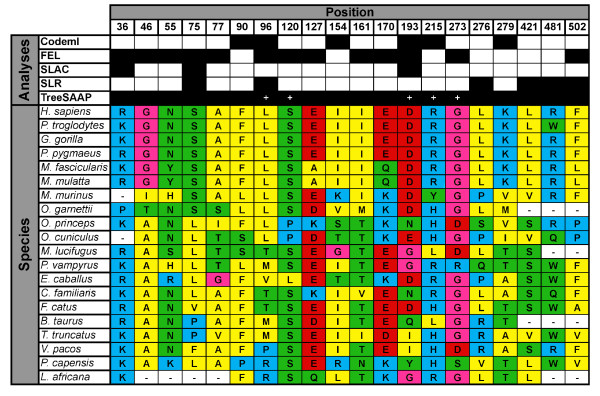
**Amino acids in the same evolutionary positions showing strong signatures of selection at the amino acids and the nucleotide level**. Sites under positive selection confirmed by the different models used in this study for the dataset of 20 species (excluding rodents). The sites were numbered according to the *Homo sapiens *position [EMBL:ENST00000361056]. The results for SLAC, FEL, PAML (Model 8), TreeSAAP (at least one property under selection) and SLR are marked with a black box in the sites showing positive selection. The sites with more than three properties under selection in TreeSAAP are marked with a white plus symbol. The background colors represent the amino acids properties: polar positive (blue), polar negative (red and green), non-polar aliphatic (yellow), and P and G (pink).

We found 69 sites with signatures of positive selection, but there was concordance between codon and amino acid level methods for only 20 of these (Figure 9). More conservatively, the number of sites dropped to five if the most stringent and conservative criterion was used (requiring three properties under selection at amino acid level to be concordant with evidence from at least one codon-based-method).

### Directed evolution analysis (DEPS)

MEPE evolution has disproportionally accumulated serines, threonines (potential phosphorylation target residues), arginines, alanines and valines, as all these amino acids showed directional evolution in the DEPS analysis (with a P-value < 0.01) (Additional file 9: Table S7). The MEPE protein had 14 sites under directional selection (Additional file 10: Table S8), seven of which are amino acids that tend to increase the disorder/unstructured probability of the regions. Additionally, eight of these 14 sites had a tendency to change to amino acids that are potentially phosphorylated residues, particularly at positions 496 and 503 (505 and 512 positions in the alignment), since these sites are relatively near the ASARM motif and the cleavage site by cathepsin-B.

### Selection Signatures and the MEPE structure

The MEPE protein belongs to a category of proteins classified as "intrinsically unstructured/natively disordered", with 53.8% and 55.8% of the human and the mouse MEPE constituted by amino acids that are associated with disorder/unstructured regions, respectively. This is reinforced given that most of the protein (around 78.8%) is disordered at a 0.05% false positive rate. Interestingly, the ASARM motif has a high content of amino acids disorder promoters while the other functional motifs (such as RGD and SGDG) incorporate regions that are structured (Additional file 11: Figure S3). The protein has a high percentage of the amino acid aspartate, which characterizes the proteins of the SIBLING family. Given the importance of disorder/order in MEPE, we analyzed the implications of selection signatures relative to the protein structural differences, and found that sites 75-Ser, 127-Glu, and 481-Arg (human MEPE as reference) are under positive selection and have a higher number of non-synonymous mutations towards codons that encode the amino acids disorder promoters.

**Figure 10:**
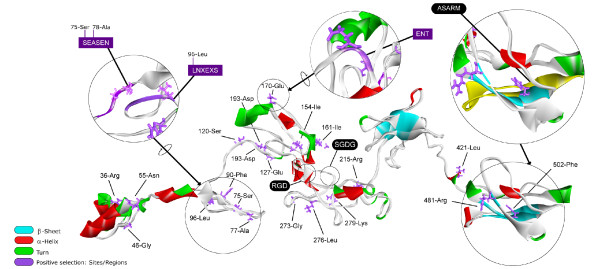
**Tertiary structure of MEPE and the positive selected sites**. The sites showing positive selection in at least two different analyses (purple sticks, three letters amino acid code) and the sites clustered showing positive selection in at least one analysis (close up circles, one letter amino acid code). The principal domains, RGD, SGDG and ASARM are also expanded and circled, as are the three "new" motifs (SEASEN, LNXEXS and ENT) showing positive selection. The secondary structure obtained is defined according to the code of colors described in the picture.

**Figure 11:**
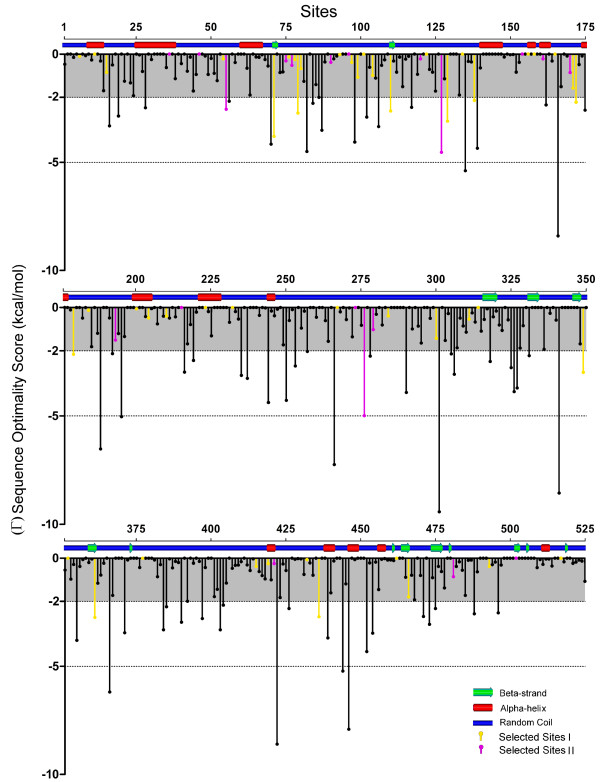
**MEPE sequence optimality scores and the secondary structure**. The sequence optimality scores (Γ) obtained in the Human MEPE, with the pink bars highlighting the sites under selection retrieved in both codon and amino acid level analyses and the yellow bars representing the sites showing selection in just one analysis (either codon or amino acid level methods). The secondary structure is represented in the top of the graph, with the nature represented: blue - random coil, green - β-Strand and red - helices.

**Figure 12:**
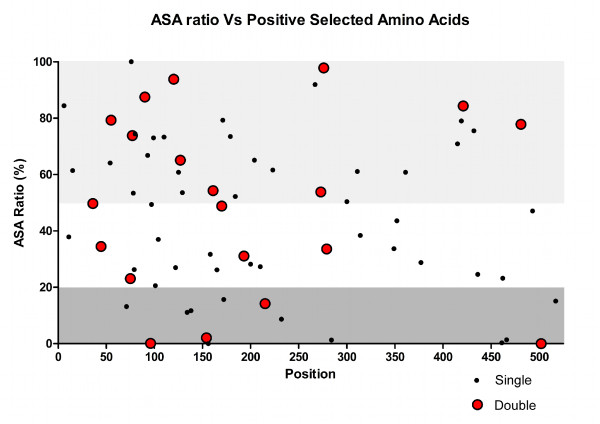
**Exposure of residues to the exterior of the MEPE protein**. Plot of the ASA ratio calculated between the side-chain and the 'random coil' value of each residue. Sites with a ratio above 50% (yellow box) are considered to be exposed to the outside of the protein whereas sites under 20% are considered to be buried (pink box). Sites under positive selection in both gene and protein-level analyses are marked with the red dots (double) and sites showing selection in at least one of the analysis is represented as black dots (single).

The tertiary structure is similar to another extracellular matrix protein, anosnim-1 [PDB:1ZLG] with a Root Mean Square Deviation (RMSD) of 5.06. To determine if the spatial organization of these sites is associated with regions of functional importance, we plotted the positively selected sites (supported by at least two different inference methods) in the tertiary structure (Figure 10). The sites showing selection signatures in both analyses are not restricted to any nature of the secondary structure (Figure 11) although most of the sites are located in random coils. In human MEPE, 69.3% of the amino acids are predicted to be found within random coils, but when this analysis is restricted to the 69 sites under positive selection (retrieved considering either the codon or amino acid level method) the percentage increases to 71%. Of the 20 sites under selection (concordant sites retrieved simultaneously with codon and amino acid level methods) the percentage increases to 75%. This shows that the sites comprehending the random coils tend to have higher chances of being under selection. Similarly, the sites under positive selection tend to be in disordered regions, as 78.8% of the MEPE protein was "intrinsic disordered". Of sites under selection in both analyses (codon and amino acid level), 90% were in disordered regions compared with 80% when considering all the sites under selection in at least one of the analyses. From the 20 sites under strong positive selection (concordant sites in both codon and amino acid level methods), 10 were solvent accessible, four were buried and the remaining six were in an intermediate category of neither buried nor exposed (Figure 12). Estimates of protein stability revealed 11 sites of human MEPE with sequence optimality values (Γ) less than -5 kcal/mol at positions 135, 166, 188, 195, 266, 301, 341, 366, 422, 444, and 446. While none of those sites correspond to a site with a signature of positive selection, when the Γ empirical cut-off is reduced to -2 kcal/mol the number of sites with a non-optimal state increases to 75. Of these, three sites are under positive selection based on both codon and amino acid analyses, and 11 of these sites show evidence of being under positive selection at either the codon or amino acid level.

## Discussion

### MEPE in the Tetrapods

Given the absence of the MEPE gene in fishes and amphibians, its origin likely coincides with the divergence of amniotes, when mineralization [11,48] and phosphate regulation [49] had a crucial role in species survival and diversification. SPP1 diverged from SPARCL1 (secreted protein acidic cysteine-rich like 1) and both are expressed in bone, participating in the bone formation (as an inhibitor of mineralization in SPP1) [50]. Therefore, the presence of SPP1 in fishes with a broader tissue expression pattern [51] suggests that SPP1 might also have similar functions to MEPE. Remarkably, after duplication, the genes were conserved during evolution and probably have differentiated to assume various functions related with tissue mineralization specificity. Recently, it was proposed that SPP1 is a more-powerful inhibitor of mineralization than MEPE [52]. This suggests that after the emergence of the complete SIBLING family in vertebrates, some functions were possibly shared among genes, notably because MEPE is absent in fishes.

The MEPE gene has similarities with other SIBLING genes, suggesting that it originated through a duplication event from another member of the gene family [5], but different dynamics of gene duplication and gene loss have occurred among lineages (e.g. absence of MEPE - Figure 1). The five genes of the SIBLING family are present in therian mammals and reptiles, but birds only have four genes (IBSP, SPP1, DMP1 and MEPE/OC-116), while fish only have two genes (SPP1 and DSPP-like). The DSPP orthology in fishes is controversial [51]. However, despite the low similarity, DSPP *starmaker *was identified as a functional orthologue [31] clearly associated with DSPP [32]. The presence/absence of various SIBLING family genes in vertebrates suggests that despite the crucial role of MEPE in mammals, birds and reptiles, its function may have been compensated in other taxa by other genes of the family. For example, in fishes a duplicated copy of SPP1 has not been described, suggesting that the fish SPP1 orthologue may have had a similar function to MEPE since SPP1 and MEPE, interact with PHEX [52]. The release of the ASARM from the MEPE protein and the phosphorylation of this motif lead to an inhibition of mineralization [48]. Similarly, the ASARM from SPP1 inhibit the mineralization [52]. Moreover, the ASARM from SPP1 is potentially phosphorylated and can interact with the hydroxyapatite crystals leading to a negative regulation of mineralization [52]. Although SPP1 has an ASARM motif near the center of the molecule, it does not have the full dentonin region (just the RGD motif). Moreover, the SPP1-ASARM has been described as a more-potent mineralization inhibitor than the MEPE-ASARM [52]. However, the knockouts of SPP1 and MEPE in mice have different phenotypes. MEPE knockouts have increased bone mass and inhibition of age-related bone loss [11] while SPP1 knockouts cause a resistance to bone loss and trabecular bone mass [53].

### Functional Conservation

The functional motifs of MEPE (RGD, SGDG and ASARM) are highly conserved among the studied mammals. In the SIBLING proteins the first coding exon encodes the signal peptides [4,54], as is observed in MEPE. The RGD motif is a common feature of all member of the SIBLING family, remaining functionally preserved after the tandem duplication that gave rise to all the members of this gene family [4]. Surprisingly, birds do not have a complete dentonin region (RGD and SGDG), although the high conservation observed among mammals suggests that this region has an important role in the function of the protein. In fact, in mammals the gene function apparently depends on the full dentonin region, as the RGD motif alone does not enhance an optimal adhesion on biomaterial surfaces in osteoblast [55]. However, when SGDG is close to RGD the mitogenic activity of dentonin increases, while the presence of only the SGDG motif promotes the cell proliferation [56]. In mammals, MEPE is involved in bone formation and osteoblast proliferation [19,56], while in birds it is involved in egg-shell formation [57]. This functional divergence may explain the sequence differences observed between the two lineages, particularly reinforced by the absence of the full dentonin region in birds. The ASARM motif is also highly conserved among mammals, but shares less than 50% similarity with the avian ASARM. Moreover, we have not detected a similar cathepsin-B cleavage site near avian MEPE-ASARM and this motif is capped at the C-terminal by 21 to 24 amino acids. Amino acids towards the C-terminal after the ASARM motif are also observed in marsupials [25]. Despite the lower similarity with the mammalian ASARM and its different position, the high conservation within birds suggests that this motif continues to have a crucial role. The changes are probably not due a relaxation of selection, but instead may have an adaptive role. In mammals, the cathepsin-B cleavage site is crucial for the function of MEPE, since this small peptide only interferes with hydroxyapaptite crystals when released [17]. Therefore, birds are also expected to have a mechanism for cleavage of ASARM. MEPE has not yet been annotated in a monotremata, no significant matches were found in a representative species of this group, the platypus (*Ornithorhynchus anatinus*). Nevertheless, the discovery of this gene in egg-laying mammals would be of great relevance to understanding the functional differences between mammals and birds.

The coding region of MEPE that flank the motifs described above is less conserved, but retain considerable phylogenetic signal across species. The human MEPE sequence has a high similarity with the great apes and with the genus *Macaca *(Cercophitecidae), even in the non-coding regions (*M. mulatta*). To a lesser degree, human MEPE also has some significant similarities in the non-coding regions with the genes of the lower primates (*M. murinus *and *O. garnetti*). MEPE appears to be particularly conserved among primates, in both coding and non-coding regions. The intronic conservation could provide valuable information about the role of non-coding sequences in the regulation/functionality of this gene. Despite the accelerated evolution in rodents, intronic conservation allowed us to reconstruct a well-supported species phylogeny from intronic sequences (even including the rodents sequences) with similar results as those obtained from MEPE coding regions.

Several human diseases increase MEPE expression [7,14,58], which may imply functional constraints in the gene even at the intronic level. Previous studies have demonstrated that highly conserved intronic regions are correlated with functional constraints and can be evidence of a hidden class of abundant regulatory elements [59]. Recently, a SNP in the region 7 kb 3' of the gene was associated with osteoporosis, a disease characterized by reduced bone mass and microarchitectural deterioration of bone tissue that reduces bone strength and leads to an increased risk of fracture [60]. These findings suggest that intergenic regions can also be important in gene function and may cause significantly different phenotypes. We hypothesize that intronic regions can also lead to significant differences at the expression level and ultimately to differences in phenotypes. This is consistent with our findings that there are strong evolutionary constraints in the MEPE intronic region.

### Selection signatures and conservation

Within mammals, MEPE in rodents is evolving faster, presenting a high amount of transitions and transversions. A similar trend is also observed in the tree shrew *T. belangeri*. However, since we only had one MEPE sequence from the order Scandentia we were unable to infer if this pattern is species-specific or if it is typical of this order. The increased number of substitutions in rodents was expected as previous studies have shown that rodents tend to accumulate more mutations in the coding regions [24,61]. We hypothesize that the observed differences in these two orders have resulted from either a divergent functional role or simply a relaxation in Darwinian selection. It is not known if the function of the rodent MEPE is similar to that in humans [62], but all the functional motifs are conserved and the signatures of positive selection or the differences observed were only detected outside of these important motifs. It is clear that positive selection may have an important role in the functional divergence of homologous proteins during adaptation to different habitats [63]. Indeed, selection may be episodic as positive and negative selection shifts over time across different lineages, reinforcing the importance of comparing sequences that have diverged within appropriate time frames [64]. The branch-site model, using rodents as foreground branches and allowing ω ratio variation not only between the branches but also among sites, identified 12 sites with strong signatures of positive selection. This suggests that the rodents and probably Scandentia may have lineage-specific selection differences in MEPE, not only in the magnitude of the selective pressure found in the branch, but also in the number of sites under selection. The acceleration of the substitution rates in rodents and the tree shrew potentially compromises the assessment of positive selection by increasing the number of synonymous mutations and because this heterogeneous site selection is observed in only two of the eight orders evaluated (i.e. Rodentia and Scandentia). The results may also be biased by the mixing of species with long and short generation times [61], as well as the related long-branch-attraction effect in phylogenetic reconstruction. Therefore, we did not include the rodents and the tree shrew in the site analysis.

The evolutionary analyses of mammalian MEPE codons (excluding the rodents and the tree shrew) found 32 sites under positive selection at codon level, and remarkably three were in functional regions of the protein, positions 6-Val and 11-Phe (Signal Peptide) and position 517-Gly (ASARM motif) (Figure 2).

Recent methods for investigating selection in protein coding genes have focused on evaluating the type of positive selection detected (directional or nondirectional, stabilizing or destabilizing), determining the presence of purifying selection, and interpreting how selection affects overall protein structure and function. Amino acid substitutions have different effects on a protein depending on differences in physicochemical properties and their position in the protein structure [65]. Here, we performed multiple analyses to differentiate among the different types of selective pressures acting in MEPE at the amino acid level. The evaluation of the amino acid physiochemical properties changes in the mammalian MEPE identified 37 more sites (36 using TreeSAAP and one using CONTEST) with selection signatures compared with the results retrieved using codon models. This shows that total reliance on models based on dN/dS using codon models may not detect some important sites with signatures of selection, often because a single adaptive mutation may occur in a small number of species, resulting in an omega lower than one. By contrast, these could also primarily be amino acid stabilizing rather than destabilizing changes, and a ω > 1 may not always be indicative of adaptive evolution.

Combining all the selection analyses, we found 69 amino acids with evidence of positive selection (20 well-supported by both codon and amino acid level approaches) (Additional file 12: Figure S4). Three clusters of positively selected sites revealed three new motifs that likely have a functional role, SEASEN (75-80), LNXEXS (96-101) and ENT (170-172) (Figure 10), using the human protein as site reference.

Selection analysis of MEPE in TreeSAAP using amino acid destabilizing properties revealed that the structural properties tend to be more affected by positive selection than the chemical properties. This suggests that the flexible and intrinsically unstructured nature of MEPE is linked to its multiple biological roles. The ASARM motif shows a "high tendency" to be a "disordered region and highly acidic", although the conformation of ASARM should be dependent on the phosphorylation level [66]. The ability to bind to hidroxyapatite is also correlated with phosphorylation state and PHEX cleavage of MEPE is dependent on the Serine phosphorylation status [8]. Moreover, our results shows that the protein tends to accumulate numerous residues with potential phosphorylation sites and this can be important to the folding/function of the protein. Proteins fold to minimize their free energy, although the structure also reflects an organization that can allow the recognition of a ligand or a transition state [67]. In fact, there is a balance between protein function and stability, and most of functional sites are non-optimal in terms of stability. If a residue is replaced by another residue, the protein activity will be reduced but the stability will be increased [67]. In MEPE we detected 75 sites with a Γ lower than -2 kcal/mol, indicating that a large number of sites in MEPE are non-optimal and therefore possibly involved in protein function. Moreover, 13 of those sites showed signatures of selection in one analysis, and sites 55, 127 and 276 in both codon and amino acid level analyses. Proteins have different secondary-structures and physicochemical properties and roles that help determine their evolutionary flexibility [68]. Thus, amino acids that comprise disordered regions, such as random coils, are more likely to be under positive selection than expected from their proportion in the proteins, compared with the residues in helices and β-structures which are subjected to less positive selection [68]. Indeed, when we compare the evidence of positive selection with the protein secondary structure in MEPE we observed that the number of sites under selection in the random coils and disordered regions are slightly higher than expected. This suggests that a high number of sites probably have a functional role or are at least relevant to an increase in MEPE protein flexibility.

Presently, most of the research on MEPE has centered on the biological role of the RGD and ASARM regions. However, our comparative study of mammalian MEPE orthologues revealed that the protein has lineage-specific properties (e.g. biochemical, evolutionary rate, intronic conservation), and that outside these two well-described motifs there are 69 sites (20 with high confidence level) under positive selection and of probable functional relevance. As positively selected sites might be either near catalytically important regions of the proteins [69] or be functionally relevant sites [70,71], these sites are good candidates for mutagenesis and structural studies to determine the functionality of MEPE relative with the other SIBLING proteins.

## Conclusions

MEPE is found in reptiles, birds and mammals (eutheria and metatheria), and to date has not been identified in monotremes. The description and study of MEPE in other taxonomic groups will be crucial to fully understanding the differences reported in avian and mammalian orthologues, and the adaptive significance of these differences. The absence of this gene in some vertebrate lineages suggests that SPP1 might partially cover the functions of MEPE in those groups. MEPE retains a strong phylogenetic signal at both coding and non-coding regions in mammals, probably due to in the functional relevance of these regions. Nevertheless, the gene is highly variable, particularly in the largest exon outside the functional motif, while other regions appear to be under strong positive selection. We found 20 sites with a significant signature of positive selection at both nucleotide and amino acid level complimentary analyses (in addition to other 69 sites with evidence of selection at either the nucleotide or the amino acid level). The analyses identified three motifs (LNXEXS, SEASEN and ENT) with selection signatures suggesting important adaptive functions. We also showed that Rodentia and Scandentia have an accelerated evolutionary rate with a unique evolutionary pattern. Finally, we showed that MEPE tends to accumulate amino acids that promote "disorder" and that present potential phosphorylation targets, supporting the contention that other regions outside the dentonin and ASARM might have crucial functional roles and demonstrating the need for future studies to understand the importance of these regions.

## Methods

### Comparative genomic analyses

MEPE nucleotide sequences were retrieved from GenBank and ENSEMBL. We aligned 26 MEPE sequences representing eight orders of mammalian species and produced two different alignments, one including all species and another excluding rodents and the tree shrew due to its nucleotide saturation bias. Given the low similarity between the avian and the mammalian sequences, the avian sequences were excluded from phylogenetic and selection analyses. BLAST searches were used to retrieve non-annotated sequences from several mammalian genomes. All the alignments were performed after the translation of nucleotides to amino acids and the corresponding alignments were back-translated to nucleotides. The alignment were performed in ClustalW [72] implemented in BIOEDIT v7.05 [73], MEGA4 [74] and LAGAN [75]. Sliding-window percent amino acid and nucleotide identity, and % GC content were calculated in Swaap 1.0.3 [76]. Saturation plots (including or excluding the third-coding position) and the estimated pI (excluding indels) were assessed in DAMBE [77]. Conservation in the coding and the non-coding regions was assessed using mVISTA [78].

### Phylogenetic analyses

We used Modelgenerator version 0.85 [79] to determine the optimal model of sequence substitution for our protein dataset, employing the Jones-Taylor-Thornton (JTT+I+G) substitution model. MrModeltest 2.3 [80] was employed to determine the optimal model of sequence substitution for our coding sequence dataset, employing the General-Time-Reversible (GTR+I+G) substitution model with the invariant site plus gamma options (five categories). Bayesian inference methods with Markov chain Monte Carlo (MCMC) sampling were performed in MrBayes [81,82]. The analysis was run for 5, 000, 000 generations with a sample frequency of 100 and burn-in was set to correspond to 25% of the sampled trees. The Maximum-Likelihood (ML) phylogenetic tree was constructed in PHYML [83], under the best-fit model for nucleotides and amino acids, 1000 bootstrap replicates and the NNI branch search algorithm. The parameters used in the tree reconstructions were set to: (i) Nucleotides: GTR+I+G with 6 substitution rate parameters and gamma-distributed rate variation with a proportion of invariant sites; (ii) Amino acids: JTT+I+G. A neighbor-joining tree was conducted in MEGA 4 [74] using the complete deletion of ambiguous data and the maximum composite likelihood option. The topologies were tested in TREE-PUZZLE [84] to identify the tree that best fits the alignment, using three tests: KH, SH and ELW. A phylogenetic signal test was performed in TREE-PUZZLE [84] using the implemented methodology [85].

### Detection of positive selection

#### Gene-level analyses

Positive selection analyses were performed in the Eutherian mammals (the closely-related taxa) to avoid nucleotide saturation and base-compositional bias. We assessed positive selection using primarily a gene-level approach [65] based on the ratio (ω) of nonsynonymous (dN) to synonymous (dS) substitutions rate (i.e., ω = dN/dS), implemented in PAML v4.3 [86] and in the web-based program SELECTON [87,88], PAML uses LRT to compare two nested models, a model that does not allow, and a model that allows, sites categories > 1 (null versus positive selection, respectively). Here, we used three LRTs based on site-specific models comparing the nested models: M1a-M2a, M7-M8 and M8a-M8. The first LRT was performed comparing M1a (nearly neutral: p0, p1, ω0 < 1, ω1 = 1, NS sites = 1) against M2a (positive selection: p0, p1, p2, ω0 < 1, ω1 = 1, ω2 < 1, NSsites = 2); the second LRT was comparing M7 (beta: p, q, NS sites = 7) with M8 (beta & ω: p0, p1, p, q, ωs > 1, NS sites = 8). The third LRT was between M8a (beta & ωs = 1: fix omega = 1, omega = 1, NS sites = 8) and M8. However, a significant LRT only demonstrated that the selection model is more suitable than the neutral model; it does not provide any indication of the sites under selection [89]. This can be accomplished through an Empirical Bayes (EB) approach to calculate the posterior probability (PP) that a given site comes from the class with ω > 1. Sites presenting a PP above the defined cut-off value (e.g. p > 95%) [90] are inferred to be under positive selection. A robust method was used to accommodate the uncertainties in the MLEs of parameters in the ω distribution, designated by BEB [90]. This approach was shown to be reliable in both small and large data sets, and also to have a good resolution power for identifying individual sites under positive selection, especially in large data sets or with strong selective pressure. We also performed an analysis using the branch-site model A [91] (model = 2 NSsites = 2), including and excluding the rodents and tree shrew as foreground branch, allowing the ω ratio vary both among sites and among lineages. The branch-site test 2 was performed using the null model, ω_2 _= 1 fixed (using the parameters fix omega = 1 and omega = 1). The sites under selection in the foreground branches were obtained after calculating probabilities of site classes using the BEB procedure.

Although the PAML models [86] allow for variation in the non-synonymous substitution rate, the synonymous rate is fixed across the sequence. To overcome that specificity, we used SLAC and FEL [92] for detecting positive selection while allowing variation in synonymous rate. SLAC is a heavily modified and improved derivative of the Suzuki-Gojobori counting approach [42,93] that maps changes in the phylogeny to estimate selection on a site-by-site basis. SLAC calculates the number of non-synonymous and synonymous substitutions that have occurred at each site using ML reconstructions of ancestral sequences [42,93]. The FEL model estimates the ratio of nonsynonymous to synonymous not assuming an *a priori *distribution of rates across sites substitution on a site-by-site analysis [93]. The SLAC and FEL methods were implemented using the web interface Datamonkey [94]. Since recombination in the gene can bias the analysis [44], we also re-run SLAC and FEL in Datamonkey using the GARD method [95], allowing each calculated partition to have its own phylogenetic tree.

Additionally, we used the LRT based analysis as implemented in the SLR (Sitewise Likelihood-Ratio) software package [45]. This method assumes that substitutions (both synonymous and non-synonymous) can occur independently with every other site, modulating substitution rates as a continuous-time Markov process. The LRT on a site-wise basis is performed testing a null model (neutrality, ω = 1) against an alternative model ω ≠ 1.

#### Protein-level analyses

We performed multiple analyses to differentiate the different types of selective pressures acting in MEPE: (i) positive versus negative selection, and (ii) stabilizing (selection that tends to maintain the overall biochemistry of the protein) versus destabilizing selection (selection that results in radical structural or functional shifts in local regions of the protein). These analyses provided insight into the structural and functional consequences of the residues under selection [46]. We used TreeSAAP v3.2 [47] and CONTEST [96] implemented in IMPACT [97] to detect selection signatures at the amino acid level. In TreeSAAP positive destabilizing-selection is detected based on the properties changes with significantly greater amino acid replacements than would be expected under neutrality for magnitude categories +7 and +8 (i.e., the two most-radical property-change categories). Within TreeSAAP, 31 amino acid properties were evaluated across the phylogenetic tree to identify the specific amino acid residues within each region that showed evidence of positive destabilization for each property. The baseml implemented PAML [86] is used in TreeSAAP [47] to reconstruct ancestral character states at the nodes on the MEPE phylogeny.

To test if evolutionary rates varied between lineages we used the relative-rate test, weighting by the predefined tree topology, as implemented in RRTree [98]. To detect directional selection over the tree or a large number of substitutions towards a particular residue in a maximum likelihood context we used the directional evolution in protein sequences (DEPS) analysis to identify statistically significant directional changes in amino acid residue frequencies [99].

#### MEPE Three-Dimensional Structure Modeling

To determine the position of the positive selected amino acids when the protein is folded, we modeled the three-dimensional (3D) structure of MEPE. Protein structure prediction can be approached in three ways: (i) comparative modeling, (ii) threading, and (iii) ab-initio folding. For MEPE, the first two methods, which build a protein model by aligning query sequences onto solved template structures, were not feasible. Thus, the only practical strategy was to run the I-TASSER [100] to obtain an ab-initio 3D model of MEPE. The model obtained using the Homo sapiens sequence had a TM Score of 0.46 ± 0.15 and a C-Score = -2.18. To accurately infer the correct topology, the model should have a C-score above -1.5, varying from [-5; 2] [100]. A TM score above 0.5 means that the obtained topology is not random [86]. Results using the sequences of the rock hyrax (out-group), the dog (i.e. one of the species showing differences in the pI) and the mouse (which demonstrates accelerated evolution) all had similar C-scores and the 3D structures similar to the results retrieved for the human MEPE, suggesting that the biochemical differences in the composition of the amino acids that constitutes the different orthologues are not imposing significant differences in the folding of the protein.

#### Structural analyses

To assess the surface exposure of the amino acids in the protein structure, we used the GETAREA 1.1 [101] web-based program based on the atom coordinates of the PDB file. This provides an estimate of the solvent exposure based on the ratio of the side-chain surface area to "random coil" value per residue, performing an analytical calculation of solvent accessible surface area residues. These are considered to be solvent exposed if the ratio value exceeds 50% and to be buried if the ratio is less than 20% [101]. Since MEPE has been described as an intrinsic unfolded protein, we also used the Protein DisOrder prediction System (PrDOS) server [102] to predict natively disordered regions of a protein chain based on the composition of the amino acid sequence. Protein stability was calculated with the PoPMuSiC 2.1 web server [103] using the MEPE PDB file previously obtained in I-TASSER to calculate the sites Γ considering all the possible mutations in each site. The secondary structure was visualized in POLYVIEW [104].

## Authors' contributions

JPM performed the phylogenetic, evolutionary and structure-function analyses and drafted the manuscript. WEJ, SJOB and VV participated in the drafting and coordination of the study. AA participated in the design, genetic analyses, drafting and coordination of the study. All authors read and approved the final manuscript.

## Supplementary Material

Additional file 1 **Table S1**. List of species used for the evolutionary genomic analyses. Click here for file

Additional file 2 **Table S2**. Tree topology and phylogenetic signal tests. Click here for file

Additional file 3 **Figure S1**. Conserved Non Coding Sequences in the 26 mammalians. Click here for file

Additional file 4 **Figure S2**. Alignment of *Homo sapiens *sequence with the three birds in the present study. Click here for file

Additional file 5 **Table S3**. Positive selection in branch-site model using rodents as foreground branch. Click here for file

Additional file 6 **Table S4**. The sites showing positive selection at codon level. Click here for file

Additional file 7 **Table S5**. The sites showing positive selection in TreeSAAP. Click here for file

Additional file 8 **Table S6**. The sites showing positive selection in CONTEST. Click here for file

Additional file 9 **Table S7**. Amino acids showing directional evolution in MEPE. Click here for file

Additional file 10 **Table S8**. MEPE protein sites showing directional evolution. Click here for file

Additional file 11 **Figure S3**. "Disordered" regions in human sequence identified using PrDOS. Click here for file

Additional file 12 **Figure S4**. Alignment of MEPE showing the functional motif and the amino acids under positive selection. Click here for file
