# Supplementary material for: Adaptive evolution of the matrix extracellular phosphoglycoprotein in mammals
Source: BMC Evol Biol. 2011 Nov 21;11:342. doi: 10.1186/1471-2148-11-342 (PMC3250972; doi:10.1186/1471-2148-11-342)
Supplement: Additional file 3 — Figure S1. Conserved Non Coding Sequences in the 26 mammalians. [file 1471-2148-11-342-S3.DOC]

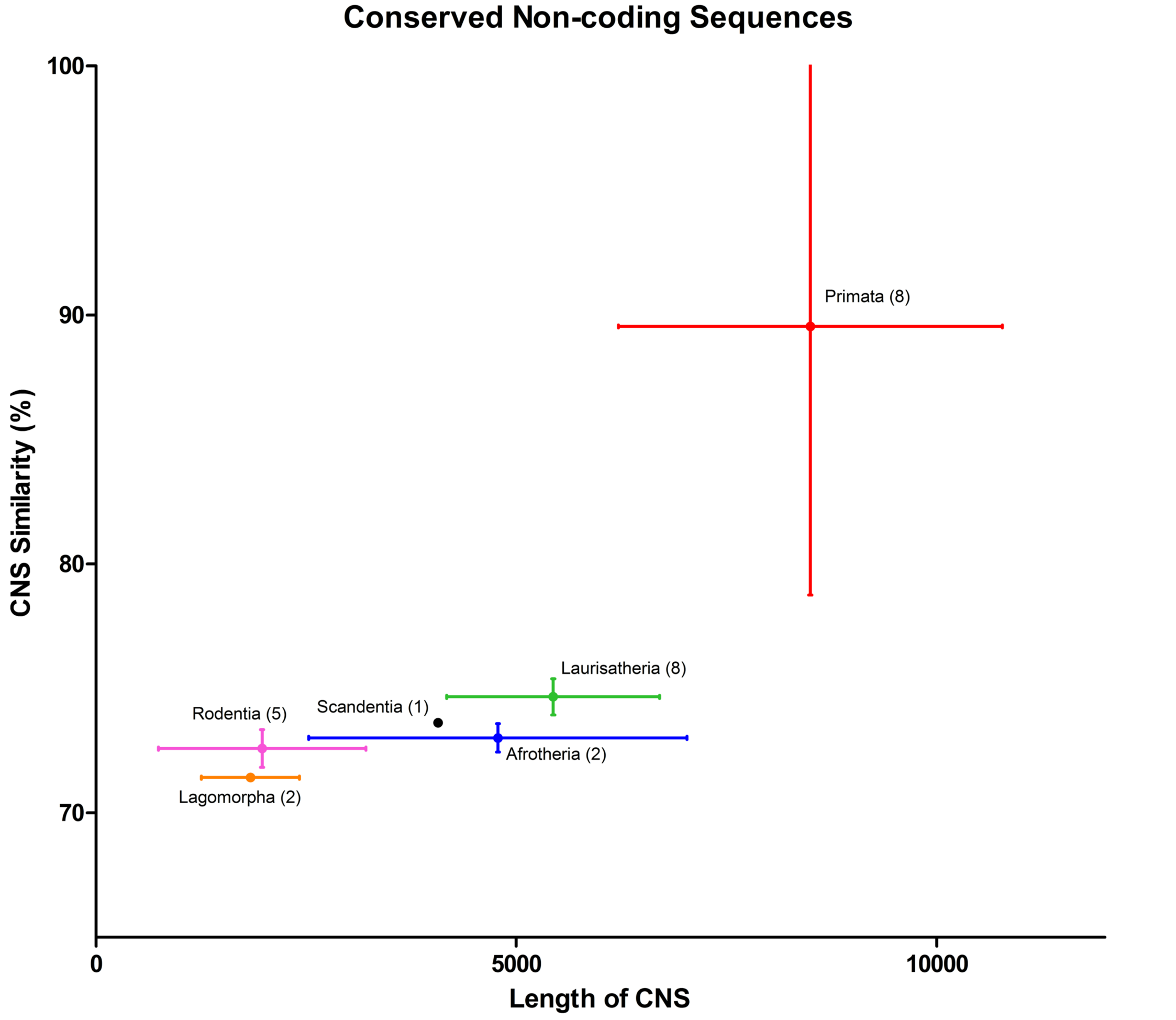


**Figure S1. Conserved Non Coding Sequences in the 26 mammalians.** Average of length and similarity of Conserved Non Coding Sequences in the 26 mammalians sequences pairwisely compared.
