# Supplementary material for: Adaptive evolution of the matrix extracellular phosphoglycoprotein in mammals
Source: BMC Evol Biol. 2011 Nov 21;11:342. doi: 10.1186/1471-2148-11-342 (PMC3250972; doi:10.1186/1471-2148-11-342)
Supplement: Additional file 4 — Figure S2. Alignment of Homo sapiens sequence with the three birds in the present study. [file 1471-2148-11-342-S4.DOC]

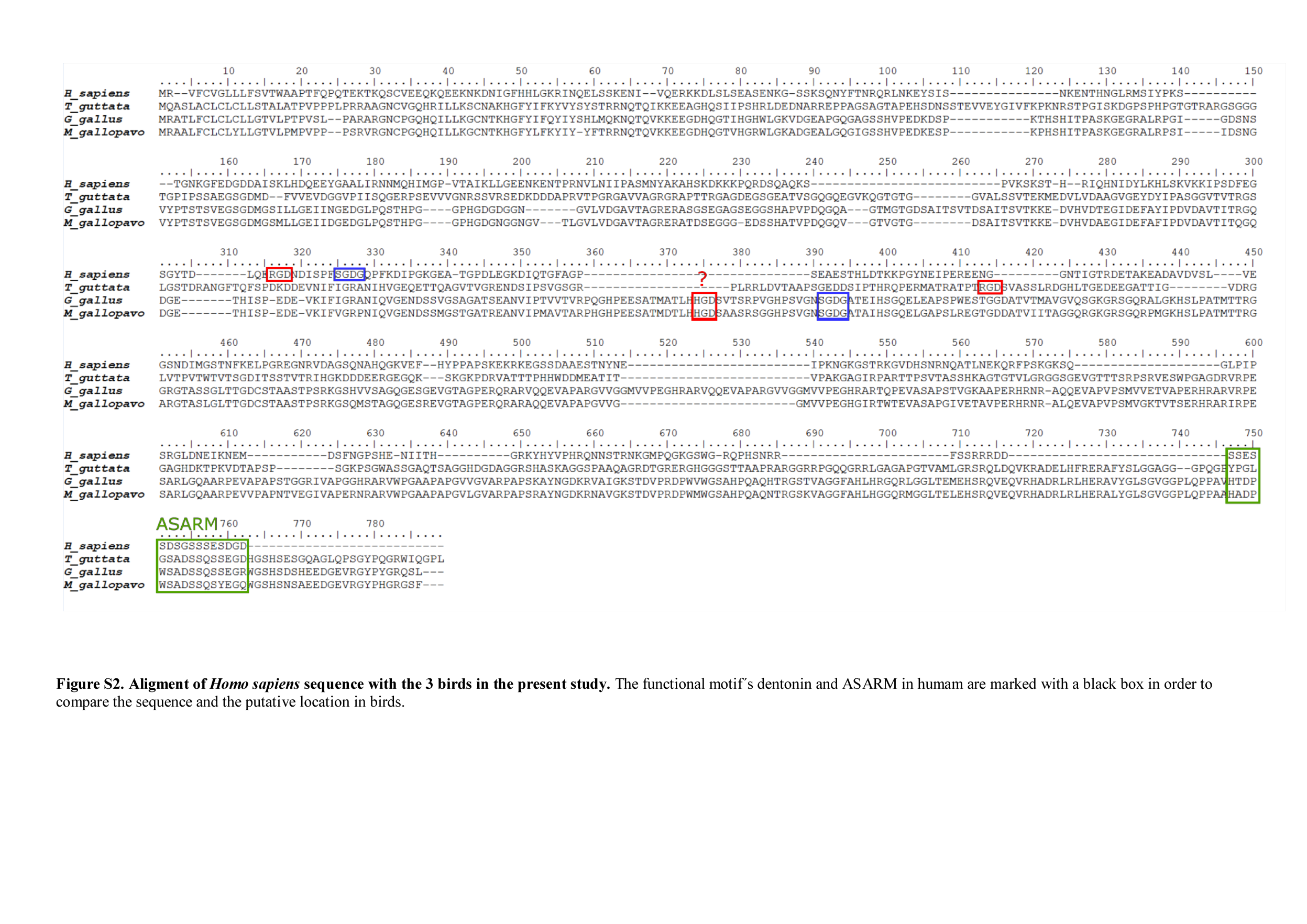


**Figure S2. Aligment of *Homo sapiens* sequence with the 3 birds in the present study.** The functional motif´s dentonin and ASARM in humam are marked within boxes in order to compare the sequence and the putative location in bird, the RGD and HGD are marked within red box, SGDG in blue box and ASARM in green box.
