# Supplementary material for: Adaptive evolution of the matrix extracellular phosphoglycoprotein in mammals
Source: BMC Evol Biol. 2011 Nov 21;11:342. doi: 10.1186/1471-2148-11-342 (PMC3250972; doi:10.1186/1471-2148-11-342)
Supplement: Additional file 11 — Figure S3. "Disordered" regions in human sequence identified using PrDOS. [file 1471-2148-11-342-S11.DOC]

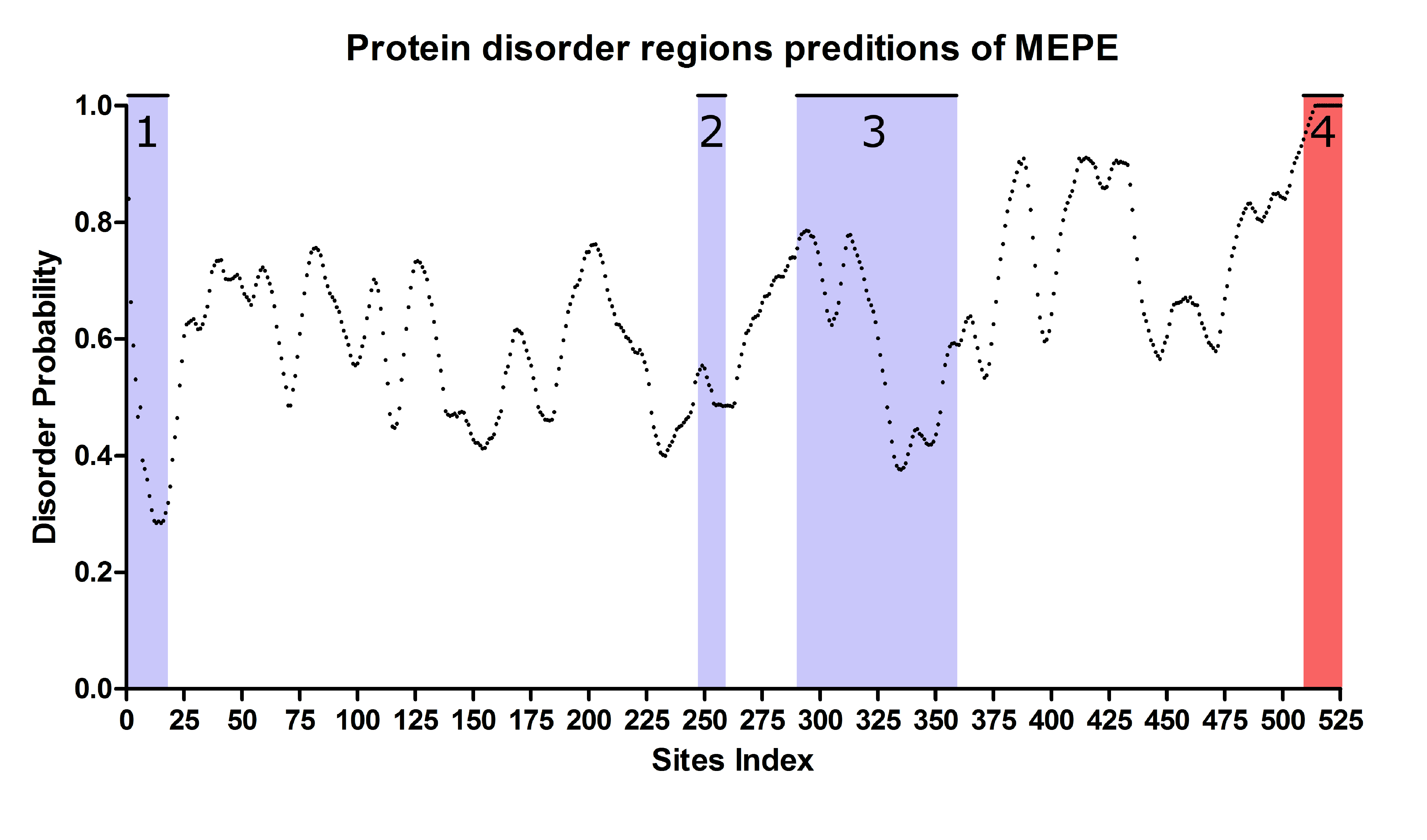


**Figure S3. “Disordered” regions in human sequence identified using PrDOS.** The regions below 0.5 are considered to be disordered residues. The locations of the main motifs are identified by shading: 1-Signal peptide, 2 –Dentonin, 3-Regulatory region, 4-ASARM motif.
