# Supplementary material for: Adaptive evolution of the matrix extracellular phosphoglycoprotein in mammals
Source: BMC Evol Biol. 2011 Nov 21;11:342. doi: 10.1186/1471-2148-11-342 (PMC3250972; doi:10.1186/1471-2148-11-342)
Supplement: Additional file 12 — Figure S4. Alignment of MEPE showing the functional motif and the amino acids under positive selection. [file 1471-2148-11-342-S12.DOC]

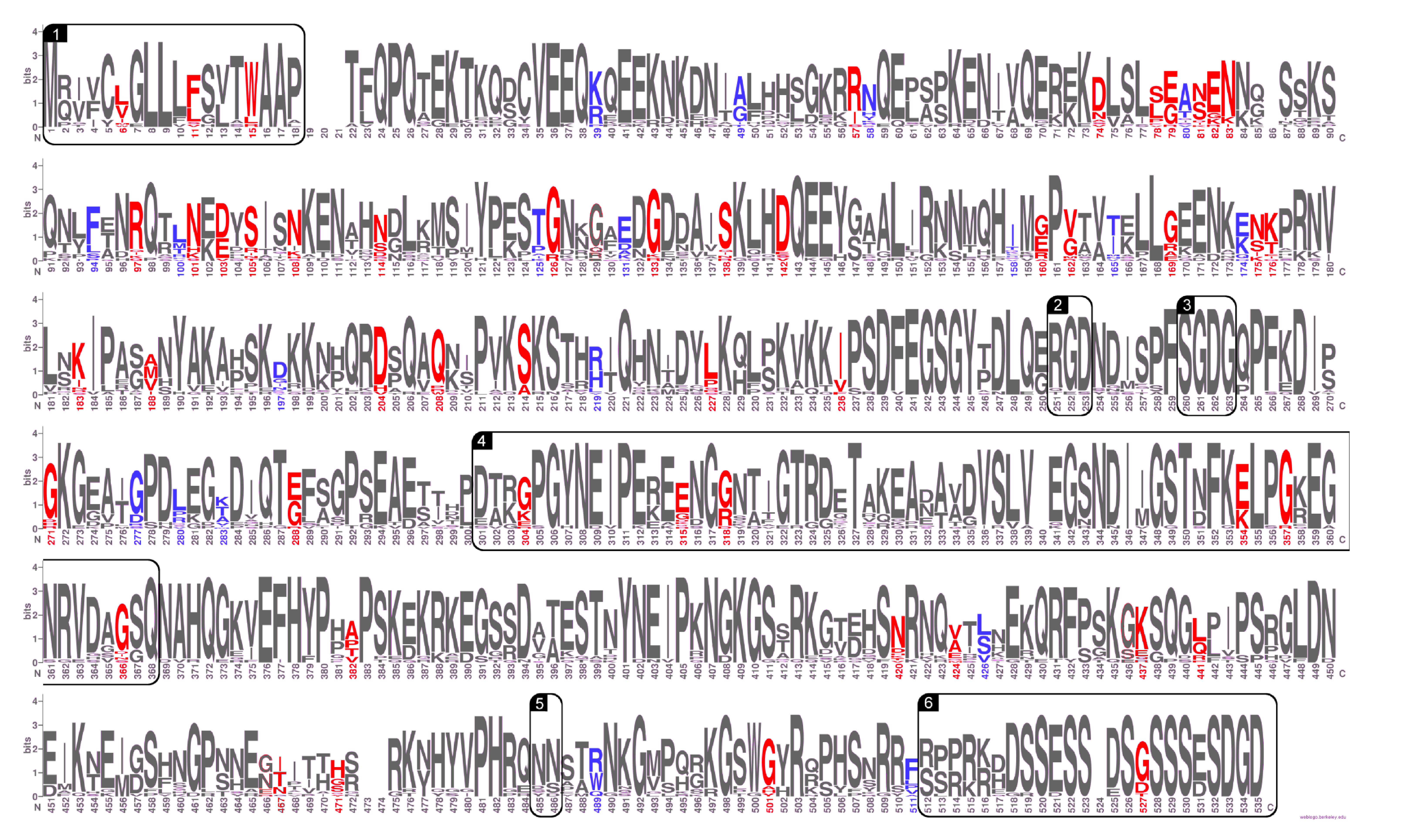


**Figure S4. Alignment of MEPE showing the functional motif and the amino acids under positive selection.** The red positions represent sites under positive selection in one analysis and blue dots represent sites under positive selection in at least two different analysis. The functional regions are inside black boxes: 1- Signal peptide, 2- RGD, 3- SDGD, 4- Regulatory region, 5 – N-glycosilation sites and 6 – ASARM.
